# Spontaneous establishing of cross-modal stimulus equivalence in a beluga whale

**DOI:** 10.1038/s41598-017-09925-4

**Published:** 2017-08-30

**Authors:** Tsukasa Murayama, Ryota Suzuki, Yurika Kondo, Mana Koshikawa, Hiroshi Katsumata, Kazutoshi Arai

**Affiliations:** 1 Tokai University, School of Marine Science and Technology, 3-20-1 Orido, Shimizu, 424-8610 Shizuoka, Japan; 2 Kamogawa Sea World, 1464-18, Higashi-machi, Kamogawa, 296-0041 Chiba, Japan

## Abstract

Beluga whales use calls to convey various information to group members. Is this communication similar to humans? We addressed this question by using the framework of stimulus equivalence. Stimulus equivalence consists of three phases: if the animal is trained to match A to B and B to C, symmetry is demonstrated by matching BA and CB, transitivity by matching AC, and equivalence by matching CA. We tested the spontaneous establishment of cross-modal stimulus equivalence between visual and auditory symbols in a beluga whale nicknamed Nack. Nack could make symmetrical choices in novel objects untrained. Moreover, visual/auditory cross-modal transitivity was formed spontaneously. Nack succeeded in six tasks, including an untrained task concerning stimulus equivalence. We conclude that Nack spontaneously formed cross-modal stimulus equivalence between visual and auditory symbols. Cross-modal stimulus equivalence was considered to exist only in humans because of linguistic faculty; however, Nack exhibited the same understanding as humans.

## Introduction

The study of animal language is one approach for investigating animal cognition. Research on language comprehension has been conducted with several species of terrestrial animals such as chimpanzees^1–8^, a gorilla^9^, an orangutan^10^, bonobos^11^ and a gray parrot^12, 13^. In these studies, visual artificial language such as gestures and lexigrams and auditory artificial language such as human speech were taught to the subjects. Though certain chimpanzees could “speak” a few human words^14^, the results were inconclusive in these language comprehension studies.

The earliest studies with marine mammals were comducted with dolphins in the 1960s by Dr. John C. Lilly^15^. He first tried to discover whether an actual dolphin language existed and attempted to teach them to speak English^16^. Schusterman and Krieger^17^, Richards *et al*.^18^, Herman^19^, and Herman *et al*.^20^ investigated language comprehension in California sea lions and bottlenose dolphins and demonstrated that these animals could partially understand the syntax or grammar of human language.

One of the cognitive characteristics useful for the emergence of language is stimulus equivalence. There are three phases in stimulus equivalence^21^; if the animal is trained to match A to B and B to C, symmetry is demonstrated by matching B to A and C to B, transitivity is demonstrated by matching A to C, and equivalence is demonstrated by matching C to A. For example, in symmetry relations, the subject that has learned to call an object apple “apple”, must be able to select an object apple when he hears the call “apple” without any additional training. In transitivity, having learned to call “apple” when the subject sees an object apple and having selected the letter “apple” when the subject hears the call “apple”, the subject must be able to select the letter “apple” when he sees an object apple without explicit training, and then, the subject must be able to select an object apple when he sees the letter “apple” without any additional training in equivalence. The acquisition of these abilities is similar to language acquisition in children^22^. Therefore, stimulus equivalence is considered an essential factor for language comprehension whereby the communicator must be able to label objects with both visual and auditory symbols. Such labeling implies an understanding of the bidirectional relationships between a representative symbol and its object. This understanding is required to establish cross-modal stimulus equivalence necessary for language comprehension. In particular, dolphins must use both audition and vision to recognize the underwater world around them through fusing these senses. Therefore, establishment of cross-modal stimulus equivalence between audition and vision is significant for dolphins and their ecology.

Studies concerning stimulus equivalence have been conducted with several terrestrial animals^23–29^. Symmetry is one of the requirements for establishing stimulus equivalence. Chimpanzee passed the symmetry test by repeating exposure of training–testing cycles on symmetry^30^. With marine mammals, Schusterman and Kastak^31^ reported that the California sea lion passed the three tests (symmetry, transitivity, and equivalence test), and succeeded in establishing spontaneous stimulus equivalence. Murayama *et al*.^32, 33^ indicated that spontaneous transitivity and symmetry were established in a beluga whale. Therefore, similar to California sea lions, the spontaneous establishment of stimulus equivalence may also develop in beluga whales.

Cross-modal stimulus equivalence is restricted to verbal language since gestural language requires only within-modal stimulus equivalence. The above mentioned studies in animal language were performed using either visual or auditory stimuli focusing on the comprehension of within-modal stimulus equivalence. However, in marine mammals, cross-modal (audition-vision) transitivity is necessary, which demonstrated with a California sea lion^34^. Cetaceans, including beluga whales, possess highly developed visual and auditory systems^35^ and make good use of them in their natural environment. Therefore, in this study, we tested the spontaneous establishment of bidirectional relationships between visual and auditory symbols to examine whether cross-modal stimulus equivalence may be established in a beluga whale.

## Methods

### Subject

The subject was a male beluga whale named Nack (body weight 879 kg, total length 384 cm, 28 years old) that was kept in Kamogawa Sea World in the Chiba prefecture in Japan. Nack underwent several kinds of cognitive experiments including matching to sample task^32, 33, 36, 37^. Nack could label some objects using sound production^38^ and also imitate human speech^39^.

### Visual stimuli

**Figure 1:**
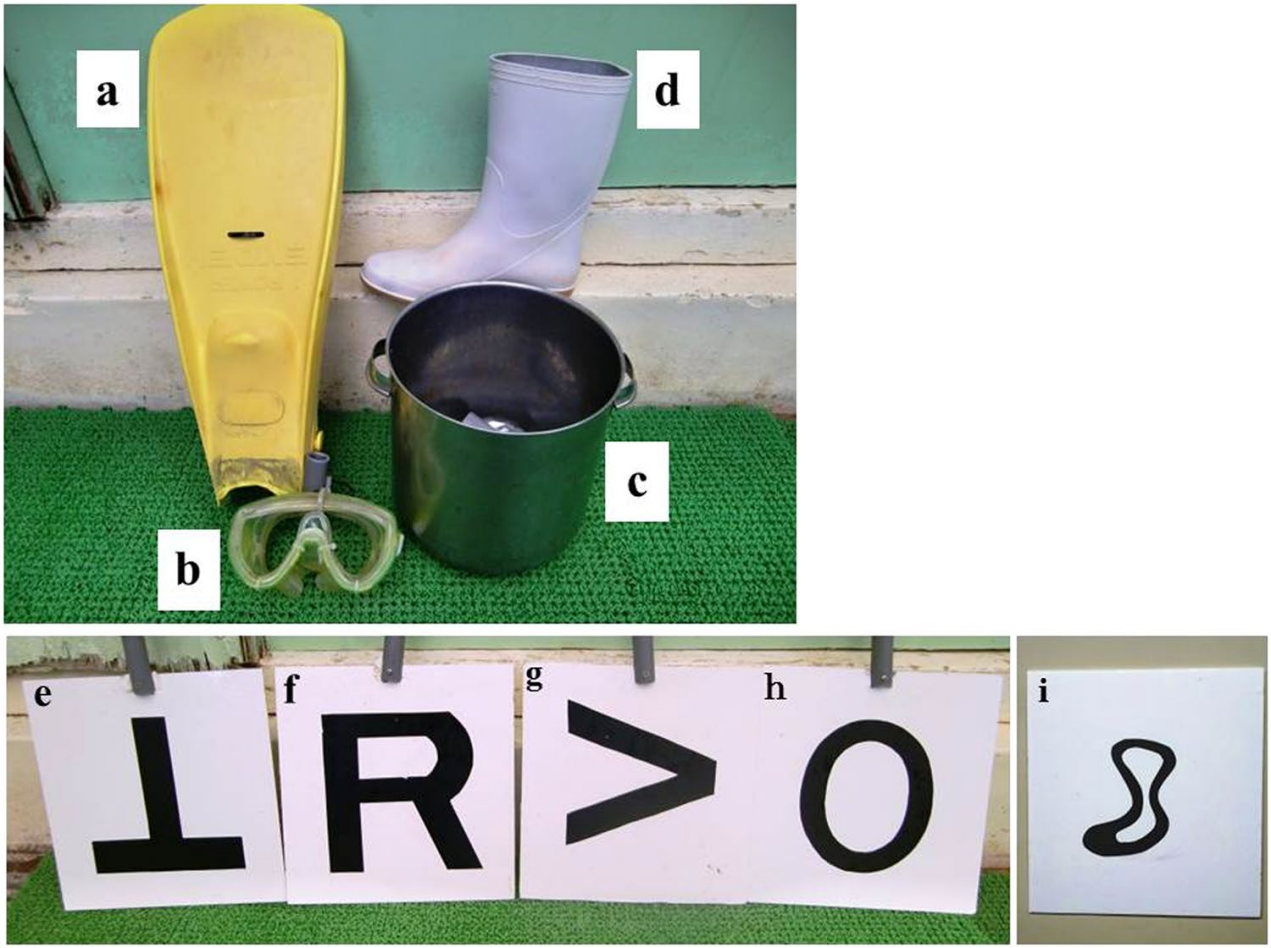
Visual stimuli. a: fin, b: mask, c: bucket, d: boot. Figure e–h correspond to fin, mask, bucket and boot, respectively. i: the design of cloud (“cloud”).

The visual stimuli (Fig. 1) were a swimming fin (hereafter “fin”), a swimming mask (hereafter “mask”), a bucket and a boot. Figures ⊥, R, >, O, and a design of a cloud (hereafter “cloud”) were drawn on boards made of vinyl chloride; these were also used as stimuli.

### Auditory stimuli

**Figure 2:**
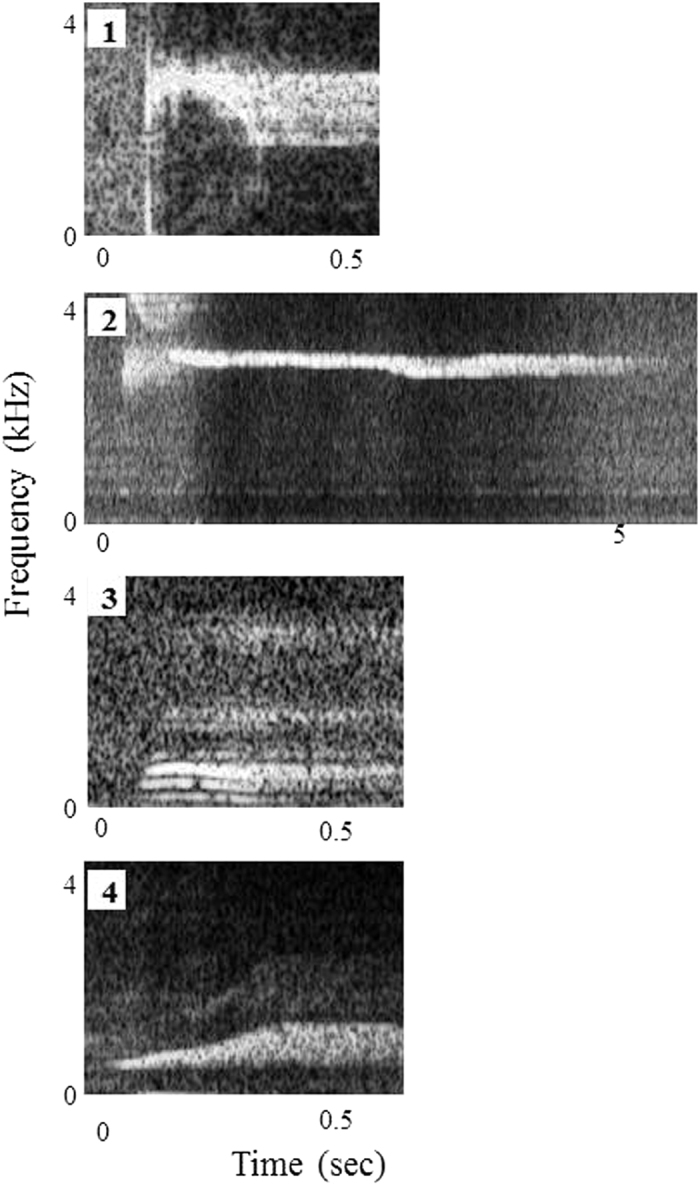
Spectrograms of calls presented to the subject as auditory stimuli. 1. fin, 2. mask, 3. bucket, 4. boot.

Since Nack realized that each object corresponded to a different call, he could emit different calls to correspond to the presented objects^38^. That is, Nack could emit a short, high-pitched sound when a fin was presented; a long high-pitched sound when a mask was presented; a short, low-pitched sound when a bucket was shown; and a short, medium-pitched sound when a boot was presented. These emitted calls were recorded and used as auditory stimuli (Fig. 2).

### Procedure

**Figure 3:**
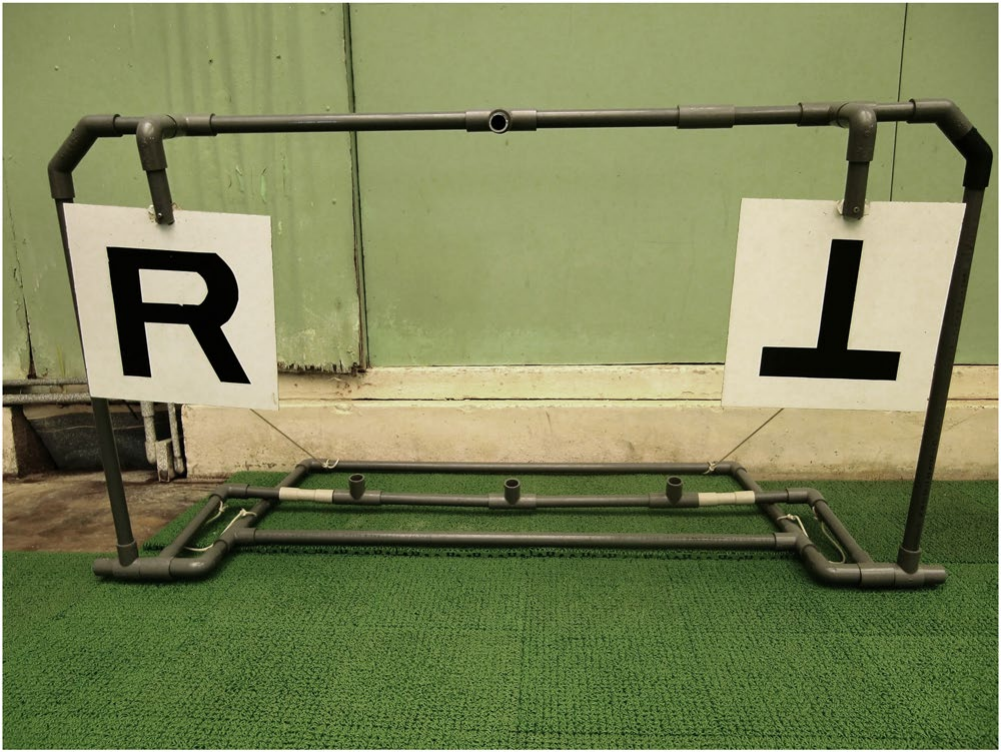
Apparatus.

The experiment was performed using a conditional discrimination task. Visual stimuli were presented to Nack by an experimenter or by an apparatus (Fig. 3). Auditory stimuli were projected through a speaker. The stimuli were presented in random order; therefore, the number of presentations of each stimulus was not uniform in each session. In a session, 10–15 trials were performed and the interval between each trial was approximately five seconds. To avoid the “Clever Hans effect”, the experimenter wore a brown-tinted goggles and the experimenter’s actions were observed by a colleague to ensure that no cues were given to the subject while performing the task.

All the research activities adhered to the Ethical Guidelines for the Conduct of Research Animals by Zoo and Aquariums issued by the World Association on Zoos and Aquariums (WAZA), the Code of Ethics issued by the Japanese Association of Zoos and Aquariums (JAZA), and the Japanese Act on Welfare and Management of Animals. All experimental protocols were approved by Kamogawa Sea World.

### Experiment 1. Spontaneous symmetry formation

**Figure 4:**
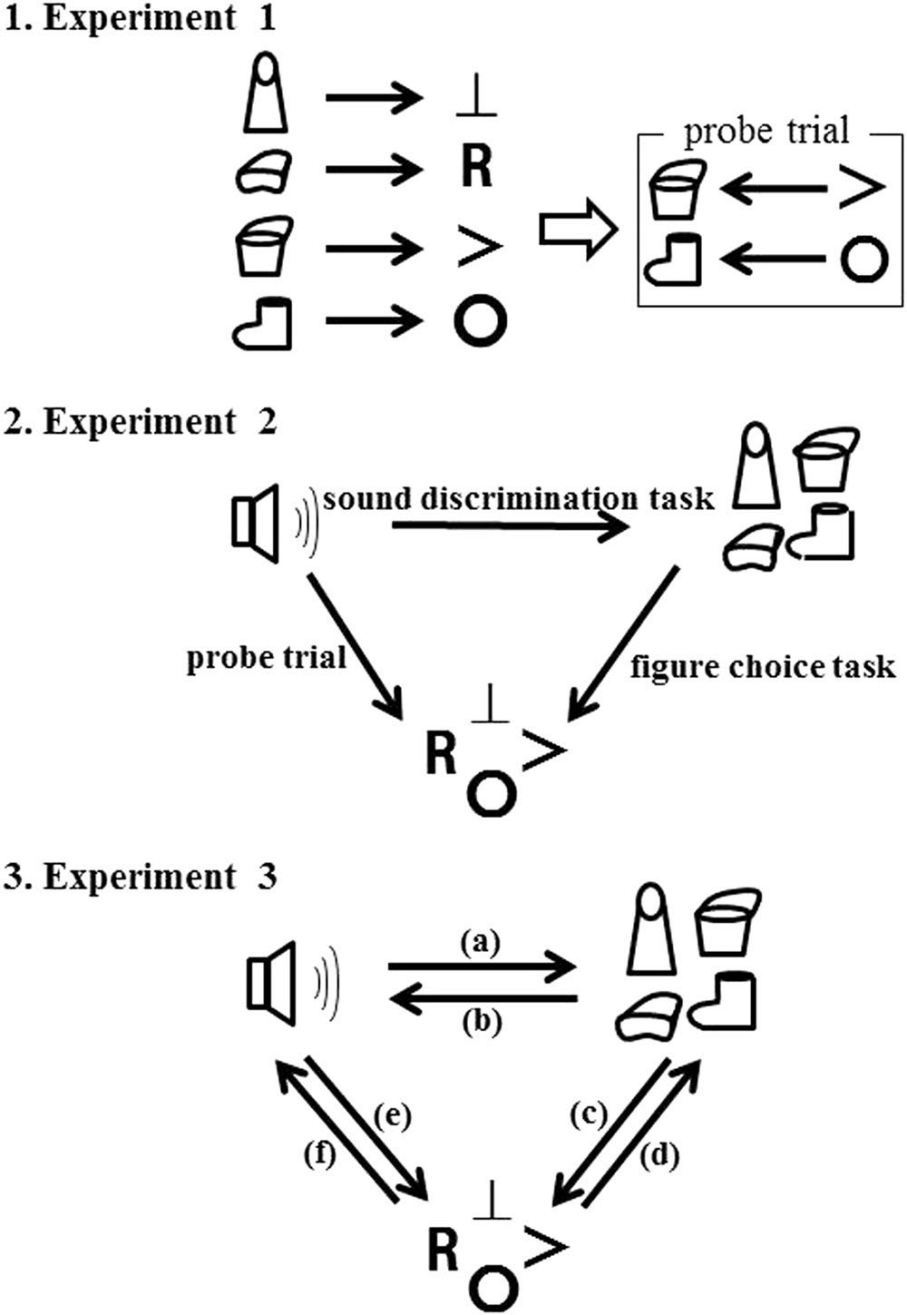
The procedure of each experiment. (Schematic diagram).

A schematic of the procedure of the experiment is presented in Figs 4-1.

During the training session, a “figure choice task” was performed. At the beginning of each trial, two of the four visual stimuli boards (Fig. 1e–h) were set in front of Nack as comparative stimuli. Next, an object was presented, and then Nack was trained to choose one of the boards (comparative stimuli) by touching the board with his snout.

The number of sample stimuli was gradually increased to avoid confusion. At first, a fin and a mask were used as sample objects. For the fin, Nack was required to select the figure ⊥, and for the mask, the figure R. These two tasks were already familiar^32, 33^ to Nack. When Nack could select each figure correctly, a bucket was shown to Nack as the sample stimulus and he was trained to select the figure > . When Nack could respond correctly to each three object, a boot was introduced as the sample stimulus in addition to the three earlier objects, and Nack was trained to choose the figure O. These two tasks were novel to Nack. These four objects were presented as sample stimulus in random order. During these four tasks, when Nack chose correctly, he was rewarded with a piece of fish. But if he chose incorrectly, no reward was given, and a 10-second time-out was introduced.

Finally, to confirm that Nack did not choose the stimuli by excluding a specific figure, one of these trained figures and a novel figure (“cloud”. Fig. 1i) were set as the comparative stimuli, and one of the four objects was presented. Nack was required to select the figure that corresponded to the sample object, and if he had chosen the stimuli by excluding a specific figure, he would have chosen the “cloud”.

In the test session, as in the baseline trial, the above mentioned “figure choice task” was conducted. One of the four objects was presented to Nack and he was ordered to select one of the two presented figures that corresponded to the sample object. In the probe (prospective randomized open blinded end-point) trial, which Nack had never experienced before, two of the four objects were presented as the comparative stimuli; then figure > or O was presented as the sample stimuli. Again, Nack was required to choose one comparative stimuli in response to the sample figure. A probe trial was performed after every 3–4 baseline trials, and Nack was not rewarded even if he selected correctly in the probe trials.

### Experiment 2. Spontaneous formation of transitivity between different mediums

Figure 4-2 shows a schematic of the experimental procedure.

In the baseline session, two tasks were performed. First, the “sound discrimination task” was carried out. Using an apparatus, two of the four objects were placed in front of Nack as comparative stimuli. Then, one of the auditory stimuli was presented, and Nack was required to select one of the two presented objects in response to the sample sound. A correct response would be to select the following objects: the fin when a short, high-pitched sound was played; the mask when a long high-pitched sound was played; the bucket when a short, low-pitched sound was played; and the boot when a short, medium-pitched sound was played. During this task, Nack was rewarded with a piece of fish for the correct responses. However, no reward was given for the incorrect responses. Nack had previously experienced this task in a previous study^38^.

Second, a “figure choice task” was carried out as the baseline task. At the beginning of each trial, two of the four stimuli figures, except for the “cloud”, were placed in front of Nack using an apparatus. Again, one of the four objects was presented, and Nack was ordered to choose the presented figure that corresponded to the sample object. The correct choices were figures ⊥, R, > , and O for the fin, mask, bucket, and boot, respectively. If Nack selected the correct object, he was rewarded with a piece of fish; however, no reward was given for incorrect responses.

In the test session, as in the baseline trial, the above mentioned two baseline tasks (“sound discrimination task” and “figure choice task”) were administered in random order. Prior to the probe trial, two of the four figures were set as comparative stimuli, and for the probe trial, one of the auditory stimuli was presented. Nack was required to select one of the two presented figures corresponding to the sample sound. A probe trial was performed after every 3–4 baseline trials. Correct responses were rewarded with a piece of fish in the baseline trials, whereas in the probe trial, no rewards were given.

### Experiment 3. Mixture of tasks

A schematic of the procedure of the tasks is presented in Figure 4-3. The following six tasks were administered in random order:

- *Sound discrimination task*: As mentioned in the baseline task of experiment 2, one of the four auditory stimuli was presented, and Nack was ordered to select one of the four objects (fin, mask, bucket, boot) that corresponded to the sample sound.
- *Different call task:* When one of the four objects (fin, mask, bucket, boot) was presented, Nack was required to emit the corresponding call for that object. As mentioned above, this task was familiar to Nack^38^.
- *Figure choice task*: As mentioned in the baseline task of experiment 2, when one of the four objects (fin, mask, bucket, boot) was presented, Nack was required to choose the corresponding figure (⊥, R, > , O).
- *Object choice task*: When one of the four figures (⊥, R, > , O) was presented, Nack was required to choose one of the objects (fin, mask, bucket, boot) presented that corresponded to the sample figure. Four associations (⊥ → fin, R → mask, >  → bucket, O → boot) were tested.
- *Sound for figure discrimination task*: As in the probe trial of experiment 2, when one of the four auditory stimuli was presented, Nack was ordered to select one of the figures (⊥, R, > , O) that corresponded to the sample sound.
- *Different call for figure task*: One of the four figures (⊥, R, > , O) was presented and Nack was required to emit the call that corresponded to the figure. This task was a novel task for Nack; he was not trained for it.

While performing these tasks, Nack was not rewarded. However, between every 3–4 trials, when the experimenter ordered him to perform some actions unrelated to the task in the study, correct behaviors were rewarded.

### Statistics

In the task of alternative choices, a binomial test was used to determine whether the percentages of correct responses were statistically significant.

## Results

### Experiment 1. Spontaneous symmetry formation

**Figure 5:**
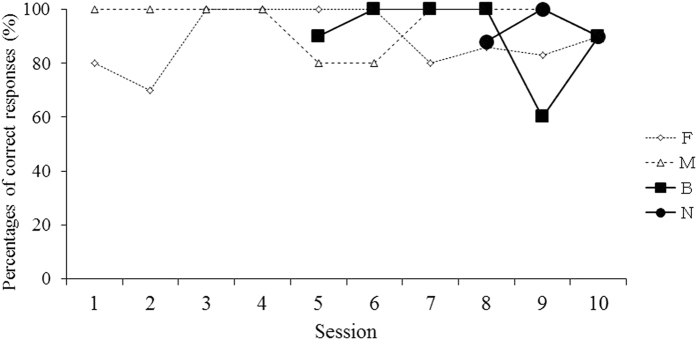
Changes in percentages of correct responses of the training session in the experiment 1. F: fin, M: mask, B: bucket, N: boot.

The changes in the percentages of correct responses in the figure choice task of the training session are shown in Fig. 5.

From session 1 to 10, a fin and a mask were used as sample stimuli. Since these two objects were familiar to Nack, he realized the relationships between the fin and mask and the correlating figures, ⊥ and R^32, 33^. Therefore, the percentages of correct responses for each object were high. After five sessions, when the bucket was added to the sample stimuli and one of these three objects was displayed in random order, the percentages of correct responses for the fin and mask remained high, and the correct responses for the bucket were also significantly high (binomial test, *p* < 0.05), except in the 9th session. After eight sessions, a boot was added to the sample objects and presented with the fin, mask and bucket. The subject successfully selected the correct figure for the boot and the percentages of correct responses were as high as they were for the other objects.

When one of those trained figures and a novel figure (“cloud”) were presented as comparative stimuli, Nack selected the correct figure perfectly. Clearly, Nack could distinguish the four objects and had learned to choose the figure that corresponded to each of them.

**Figure 6:**
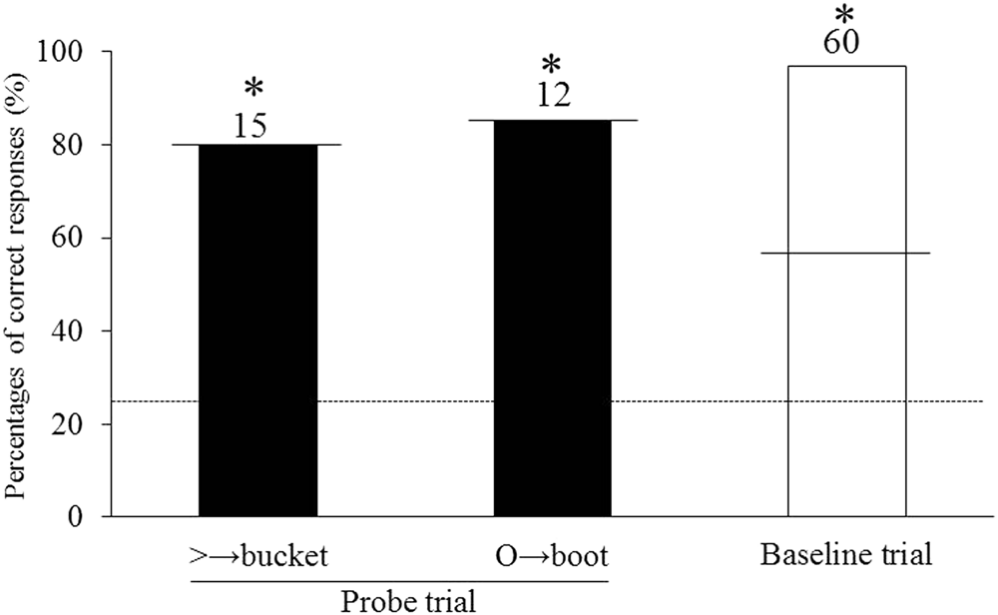
Percentages of correct responses of the test session in the experiment 1. Each numeral means the number of trials. Dashed line means chance level and solid lines mean significance level (*significant, *p* < 0.05).

The percentages of correct responses for the test session are shown in Fig. 6. In the baseline trial, correct choices reached a level of significance (42%, binomial test, *p* < 0.05). In the probe trial, with the figures > and O, the correct choices of the bucket and the boot respectively were made without any confusion. Nack responded correctly from the first trial in the probe test. The percentages of correct responses for both figures were high (80% for figure > , 83% for figure O) and exceeded the significance level of *p* < 0.05 (60% for bucket and 58% for boot) of a binomial test. There were no significant differences between the correct percentage on baseline trial and that on each probe trial. These results demonstrate that Nack could make a symmetrical choice untrained.

### Experiment 2. Spontaneous formation of transitivity between different mediums

**Figure 7:**
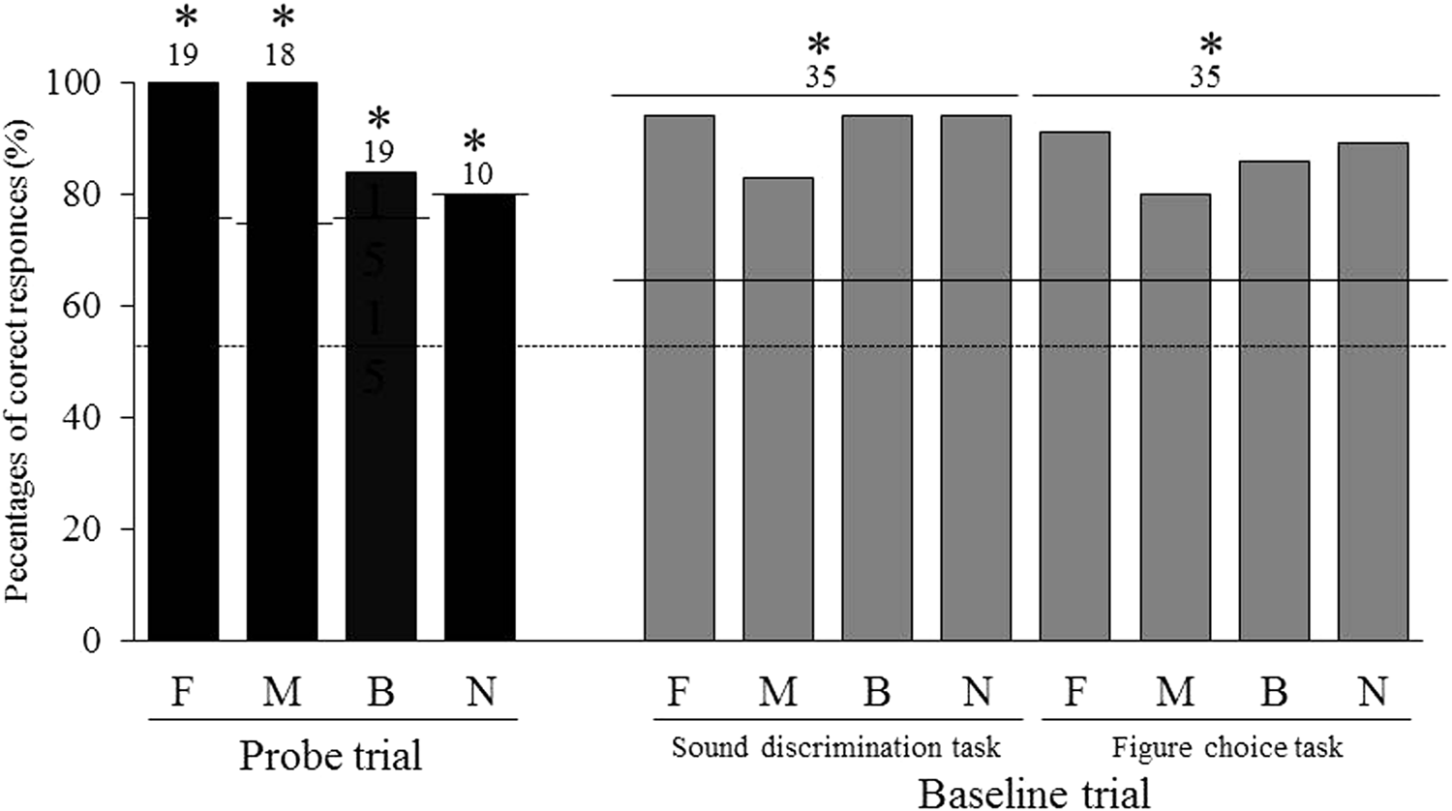
Percentages of correct responses of the experiment 2. F: fin, M: mask, B: bucket, N: boot. Each numeral means the number of trials. Dashed line means chance level and solid lines mean significance level (*significant, *p* < 0.05).

The percentages of correct responses in the baseline and probe trial test sessions are shown in Fig. 7.

In the baseline session, Nack correctly selected objects that corresponded to the sample sounds in the “sound discrimination task”. This was also true for the “figure choice task”. In the probe trial, when the four sample sounds were presented in random order, Nack made correct figure choices that corresponded to the sample sound without any confusion, even though he was not rewarded at all. Nack responded correctly from the first trial in the probe test. The percentages of correct responses was 100% for the fin and the mask, 84% for the bucket, and 80% for the boot, all of which exceeded the significance level of *p* < 0.05 (binomial test), implying that Nack spontaneously developed transitivity. There were no significant differences between the correct percentage on the baseline trial and that on each probe trial.

### Experiment 3. Mixture of tasks

**Figure 8:**
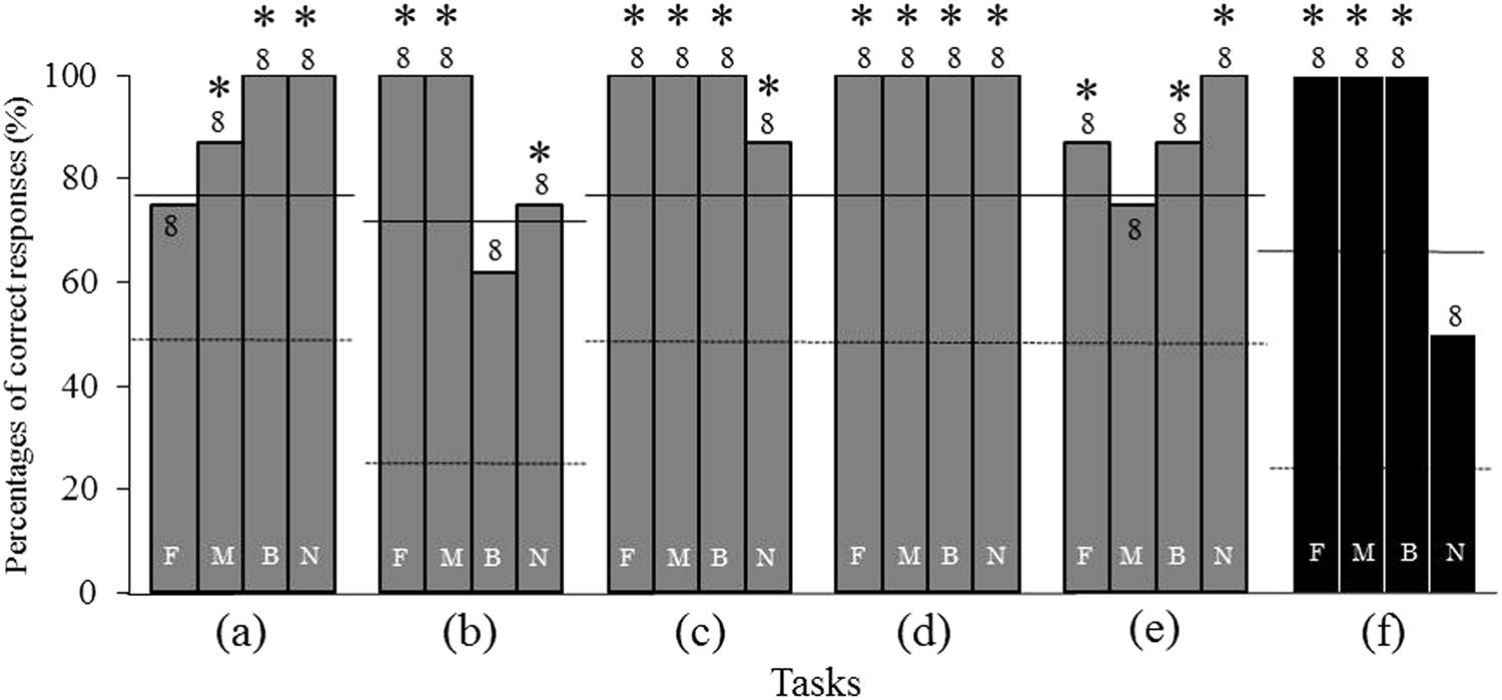
Percentages of correct responses of the experiment 3. (**a**) Sound discrimination task, (**b**) Different call task, (**c**) Figure choice task, (**d**) Object choice task, (**e**) Sound for figure discrimination task, (**f**) Different call for figure task. The task (**f**), Different call task, is novel to the subject. Each numeral means the number of trials. F: fin, M: mask, B: bucket, N: boot. Dashed lines mean chance level and solid lines mean significance level (*significant, *p* < 0.05).

Six tasks were performed in random order and the percentages of correct responses for each task are shown in Fig. 8.

In most of the tasks, Nack responded correctly and the percentages of correct choice were significantly high (binomial test, *p* < 0.05) or much higher than a chance level in every task. This indicates that Nack could respond correctly even when the tasks were assigned in random order.

What is especially notable is that, even though Nack had never experienced task (f), the different call for the figure task, he could emit the correct call to the presentation of figures ⊥, R, > and O, with the percentages of correct responses exceeding significant levels (75%, binomial test, *p* < 0.05) or a chance level. These results suggest that Nack spontaneously understood which call was to be emitted even being untrained.

## Discussion

In the test session, Nack was not rewarded regardless of which comparisons he chose. With this procedure, there is no learning effect.

In the “figure choice task”, Nack succeeded in selecting the correct figures corresponding to the novel objects. In these trials, the control by the relations between the sample objects and the positive comparison stimuli (S+control)^40^ was formulated. In other words, the subject selected the familiar (positive) stimulus out of a set of comparisons, namely, novel (“cloud”) and familiar ones (⊥, R, > , O) in the presence of the sample objects. However, when the bucket and the boot were added to the sample as novel stimuli, another type of control, control by exclusions^40^, was formulated. That is, these sample objects were novel (undefined), the subject chose the comparison by excluding the comparison that was defined as a familiar object. However, the subject finally responded correctly in the random presentation of these four objects, indicating that he chose the correct figures by matching the sample object with the figure, not by matching by excluding the familiar figure.

Several species such as primates^27, 28^ and pigeons^23, 26^ have failed to pass the symmetry test. Since Nack had been tested on symmetrical relations using a fin and a mask before this study^32^, responses could be expected to be correct. However, he had no previous experience of the test using a bucket or a boot; yet, he was still able to respond correctly to the novel stimuli. Thus, in this beluga whale, the capacity to select a bucket and a boot can be taken as evidence for the capacity to identify symmetrical relationships for novel objects. Schusterman and Kastak^31^ also demonstrated that symmetry was formed spontaneously in a California sea lion by performing the symmetry test using various stimuli. Schusterman suggested that the reason the sea lion passed the symmetry test was that the subject had experienced many stimuli on symmetry trials. Since Nack had participated in various cognitive studies before this study^32, 33, 36–39^ and had also passed the symmetry tests for a fin and a mask before^32^, it is possible that he already possessed the concept of symmetry^41^ prior to this study. Such experiences may have contributed to the subject’s success in the symmetry test on novel objects (bucket and boot) in the present study.

The spontaneous establishment of cross-modal stimulus equivalence is natural in human children, but quite rare in non-human animals. In tests (a) and (b), since Nack had been already trained to emit a vocalization upon matching visual stimuli, this trained conversion from heard to vocalized stimuli would have affected Nack’s performance.

Concerning transitivity, several kinds of species have passed the within-modal transitivity test^31, 33, 42–44^. Since echoic/visual cross-modal performance in bottlenose dolphin has been reported^45, 46^, the ability of cross-modal recognition is thought to also exist in other marine mammals such as the California sea lion^34^. In our study, a cross-modal transitivity test was carried out, and Nack was able to pass the test. Consequently, a spontaneous visual/auditory cross-modal transitivity was formed.

Finally, when all of the tasks were mixed, Nack responded correctly to most of them, including the untrained task. It appears that the relationship of transitivity was formed spontaneously even in untrained tasks, by extrapolating from the two former tasks, (d) Object choice task and (b) Different call task. In tasks (e) and (f), symmetry was established both in the comprehension aspect and the production aspect of the auditory stimulus.

In both the symmetry and the transitivity tests, Nack responded correctly from the first trial of each session, and he was not rewarded during the probe test. Therefore, it is suggested that symmetry was established prior to the session, not during the session.

Nack passed the symmetry test among uni-modal stimuli in the previous study^32^, therefore, the symmetry established among uni-modal stimuli appears to have been transferred to cross-modal stimuli in the present study.

Nack succeeded in these six tasks, including the untrained task, indicating that bidirectional relationships or associations among all of the samples and comparisons were learned in the trained tasks and spontaneously produced in the untrained task. We conclude that Nack formed cross-modal stimulus equivalence spontaneously. This ability was previously considered to exist only in linguistic humans; however, our beluga whale, Nack, exhibited the same understanding/ability to use rules of logic as linguistic humans do.

The ecology of an animal affects the establishment of complex relationships between stimuli. Animals perceive an object through several kinds of sensory systems. In particular, social animals have to identify family members, enemies, and rivals by making good use of those sensory cues, as they must recognize them by combining all the information obtained from those sensory systems^47, 48^. Stimulus equivalence allows other relationships to be established spontaneously without any training if a specific relationship is recognized^21^. Therefore, fusing a variety of sensory cues contributes to the make-up of stimulus equivalence. Highly socialized animals including the beluga whale can readily recognize social relationships between individuals based on their development of equivalence classes.

We conclude that Nack was able to label four objects with sounds (his calls) and specific figures because cross-modal stimulus equivalence was formed spontaneously.

## References

[1] Gardner RA, Gardner BT (1969). Teaching sign language to a chimpanzee. Science.

[2] Gardner, R. A., Gardner, B. T. & Drumm, P. *Teaching sign language to chimpanzees* (University of New York Press, New York, 1989).

[3] Premack AJ, Premack D (1972). Teaching language to an ape. Scientific American.

[4] Rumbaugh, D. M. *Language learning by a chimpanzee* (Academic Press, New York, 1977).

[5] Asano T (1982). Object and color naming in chimpanzees (*Pan troglodytes*). Proceedings of Japan Academy.

[6] Savage-Rumbaugh ES, Rumbaugh DM, Smith ST, Lawson J (1980). Reference: The linguistic essential. Science.

[7] Matsuzawa T (1985). Color naming and classification in a chimpanzee (*Pan troglodytes*). Journal of Human Evolution.

[8] Savage-Rumbaugh, E. S. *Ape Language: From Conditioned Response to Symbol* (Columbia University Press, New York, 1986).

[9] Patterson F (1978). The gesture of a gorilla: Language acquisition in another Pongid. Brain and Language.

[10] Miles, H. L. The cognitive foundations for reference in a signing orangutan. *Language and intelligence in monkeys and apes: Comparative developmental perspectives* (Parker, S. T. & Gibson, K. R. (eds)) 511–539 (Cambridge University Press, Cambridge, 1990).

[11] Savage-Rumbaugh, E. S. *Kanzi: A Most Improbable Ape*. (NHK Publishing, Tokyo, 1993).

[12] Pepperberg IM (1990). Cognition in an African gray parrot (*Psittacus erithacus*): Further evidence for comprehension of categories and labels. Journal of Comparative Psychology.

[13] Pepperberg, I. M. *The Alex Studies*. (Harvard University Press, Cambridge, 1999).

[14] Hays, C. *The ape in our House* (Harper, New York, 1951).

[15] Lilly, J. C. *Man and Dolphin: Adventures of new scientific frontier*. (Doubleday, New York, 1961).

[16] Lilly, J. C. *The mind of the dolphin* (Doubleday, New York, 1967).

[17] Schusterman RJ, Krieger K (1984). California sea lion are capable of semantic comprehension. The Psychological Record.

[18] Richards DG, Wolz JP, Herman LM (1984). Vocal mimicry of computer generated sounds and vocal labeling of objects by a bottlenosed dolphin. Tursiops truncatus. Journal of Comparative Psychology.

[19] Herman, L. M. Cognition and language competencies of bottlenosed dolphins. *Dolphin cognition and behavior: A comparative approach* (Schusterman, R. J., Thomas, J. & Wood, F. J. (eds)) 221–251 (Lawrence Erlbaum, Hillsdale, 1986).

[20] Herman LM, Richards DG, Wolz JP (1984). Comprehension of sentences by bottlenosed dolphins. Cognition.

[21] Sidman M, Tailby W (1982). Conditional discrimination vs. matching-to-sample: An expansion of the testing paradigm. Journal of the Experimental Analysis of Behavior.

[22] Mazur, J. E. *Learning and Behavior*. (Prentice Hall, NJ, 1994).

[23] Lipkens R, Kop PFM, Matthijs W (1988). A test of symmetry and transitivity in the conditional discrimination performances of pigeons. Journal of the Experimental Analysis of Behavior.

[24] Sidman M (1982). A search for symmetry in the conditional discriminations of rhesus monkeys, baboons, and children. Journal of the Experimental Analysis of Behavior.

[25] Manabe K, Kawashima T, Staddon JER (1995). Differential vocalization in budgerigars: Towards an experimental analysis of naming. Journal of the Experimental Analysis of Behavior.

[26] Davis, H. Logical transitivity in animals. *Cognitive aspects of stimulus control*. (Honig, W. K. & Fetterman, J. G. (eds)) 405–429 (Psychology Press, Hillsdale, 1992).

[27] D’Amato MR, Salmon DP, Loukas E, Tomie A (1985). Symmetry and transitivity of conditional relations in monkeys (*Cebus apella*) and pigeons (*Columba livia*). Journal of the Experimental Analysis of Behavior.

[28] Sidman M (1982). A search for symmetry in the conditional discriminations of rhesus monkeys, baboons, and children. Journal of the Experimental Analysis of Behavior.

[29] Tomonaga M, Matsuzawa T, Fujita K, Yamamoto J (1991). Emergence of symmetry in as visual conditional discriminations by chimpanzees (*Pan troglodytes*). Psychological Reports.

[30] Yamamoto J, Asano T (1995). Stimulus equivalence in a chimpanzee (*Pan troglodytes*). The Psychological Record.

[31] Schusterman RJ, Kastak DA (1993). California sea lion (*Zalophus californianus*) is capable of forming equivalence relations. The Psychological Record.

[32] Murayama T (2008). Formation of symmetry in beluga. Cognitive Studies.

[33] Murayama T, Tobayama T (1997). Preliminary study on stimulus equivalence in beluga (*Delphinapterus leucas*). The Japanese Journal of Animal Psychology.

[34] Lindemann- Biolsi KL, Reichmuth C (2014). Cross-modal transitivity in California sea lion (*Zalophus californianus*). Animal Cognition.

[35] Herman, L. M. Cognitive characteristics of dolphins. *Cetacean behavior: mechanisms and functions* (Herman, L. M. (ed.)), 363–427 (Wiley Interscience, New York, 1980).

[36] Murayama, T. & Tobayama, T. Preliminary study of mental rotation in beluga. *Abstract of the XXIV International Ethological Conference*, Hawaii, USA; 114 (1995).

[37] Murayama T, Iochi A, Tobayama T (2001). Discrimination of ellipse from circle in white whale. Nippon Suisan Gakkaishi.

[38] Murayama T (2012). Preliminary study of object labeling using sound production in a beluga. International Journal of Comparative Psychology.

[39] Murayama T, Iijima S, Katsumata H, Arai K (2014). Vocal imitation of human speech, synthetic sounds and beluga sounds, by a beluga (*Delphinapterus leucas*). International Journal of Comparative Psychology.

[40] Tomonaga M (1993). Tests for control by exclusion and negative stimulus relations of arbitrary matching to sample in a symmetry emergent chimpanzee. Journal of the Experimental Analysis of Behavior.

[41] Sidman, M. *Equivalence relations and behavior: A research story*. (Authors Cooperative, 1994).

[42] McGonigle B, Chalmers M (1977). Are monkeys logical?. Nature.

[43] Treichler FR, Van Tilburg D (1996). Concurrent conditional discrimination tests of transitive inference by macaque monkeys: List linking. Journal of Experimental Psychology: Animal Behavior Processes.

[44] Davis, H. Logical transitivity in animals. *Cognitive aspects of stimulus control*. (Honig, W. K. & Fetterman J. G. (eds)) 405–429 (Lawrence Erlbaum, Hillsdale, 1992).

[45] Harley, H. E. Xitco, M. J. Jr. & Roitblat, H. L. Echolocation,cognition, and the dolphin7s world. *Sensory Systems of Aquatic Mammals* (Kastelein, R. A., Thomas, J. A. & Nachtigall, P. A. (eds)) 529–542 (De Spil Publisher, Woerden, 1995).

[46] Herman, L. M. & Pack, A. A. Echoic visual cross modal recognition by a dolphin. *Marine Mammal Sensory Systems* (Kastelein, R. A., Thomas, J. A. & Nachtigall, P. A. (eds)) 709–726 (Plenum Press, New York, 1992).

[47] Schusterman RJ, Kastak D (1998). Functional equivalence in a California sea lion: relevance to animal social and communicative interactions. Animal Behaviour.

[48] Schusterman RJ, Reichmuth CJ, Kastak D (2000). How animals classify friends and foes. Current Directions in Psychological Science.

